# Delineation of lung cancer with FDG PET/CT during radiation therapy

**DOI:** 10.1186/s13014-018-1163-2

**Published:** 2018-11-12

**Authors:** J. Ganem, S. Thureau, I. Gardin, R. Modzelewski, S. Hapdey, P. Vera

**Affiliations:** 1 Nuclear Medicine Department, Henri Becquerel Cancer Centre and Rouen University Hospital, Rouen, France; 2 QuantIF-LITIS (EA [Equipe d’Accueil] 4108-FR CNRS [Fédération de Recherche-Centre National pour la Recherche Scientifique] 3638), Faculty of Medicine, University of Rouen, Rouen, France; 3 Department of Radiotherapy and Medical Physics, Henri Becquerel Cancer Centre and Rouen University Hospital, Rouen, France

**Keywords:** Delineation, Radiation therapy, PET/CT, Lung cancer, perPET-RT

## Abstract

**Objectives:**

To propose an easily applicable segmentation method (perPET-RT) for delineation of tumour volume during radiotherapy on interim fluorine 18 fluorodeoxyglucose (FDG) positron emission tomography/computed tomography (PET/CT) in patients with non-small cell lung cancer (NSCLC).

**Material and methods:**

Sixty-seven patients (51 primary tumours, 60 lymph nodes), from 4 prospective studies, underwent an FDG PET/CT scan during the fifth week of radiation therapy, using different generations of PET/CT. Per-therapeutic PET/CT scans were delineated in consensus by two experienced physicians leading to the gold standard threshold to be applied. The mathematical expression of Th_opt_, the optimal threshold to be applied as a function of the maximum standard uptake value (SUV_max_), was determined. The performance of this method (perPET-RT) was assessed by computing the DICE similarity coefficient (DSC) and was compared with 8 fixed threshold values and 3 adaptive thresholding methods.

**Results:**

Th_opt_ verified the following expression: Th_opt_ = A.ln(1/SUV_max_) + B where A and B were 2 constants. A and B were independent from the generation of PET/CT, but depended on the type of lesions (primary lung tumours vs. lymph nodes). PerPET-RT showed good to very good agreement in comparison to the gold standard. The mean and standard deviation of DSC value was 0.81 ± 0.13 for lung lesions and 0.78 ± 0.15 for lymph nodes. PerPET-RT showed a significant better agreement than the other segmentation methods (*p* < 0.001), except for one of the adaptive thresholding method ADT (*p* = 0.11).

**Conclusion:**

On the database used, perPET-RT has proven its reliability and accuracy for tumour delineation on per-therapeutic FDG PET/CT using only SUV_max_ measurement. This method may be used to delineate tumour volume for dose-escalation planning.

**Trial registration:**

NCT01261598, NCT01261585, NCT01576796.

## Introduction

Non-Small Cell Lung Cancer (NSCLC) represents a leading cause of death by cancer in the world, especially in Europe and North America. Treatment modalities should be personalized according to the patient’s clinical condition, tumor staging, histological/molecular profile, whether disease is resectable, locally advanced or advanced and may comprise surgery, radiation therapy and chemotherapy [1–3].

FDG PET/CT (^18^F-fluorodeoxyglucose positron emission tomography/computed tomography) has proven utility to accurately to delineate the tumour volume for external radiation therapy [4, 5]. In the case of NSCLC, pre-therapeutic FDG PET/CT allows the delineation of the metabolic tumour volume (MTV), the exclusion of non-tumoral abnormalities (such as atelectasis) and also improves inter and intra observers reproducibility [6, 7], which are one of the main limitations when delineating on CT modality alone.

Several radiation therapy strategies have been considered so far Bradley et al. showed that high dose conformational radiation therapy was not better than standard-dose radiation therapy and even potentially harmful, due to increased toxicity [8].

Current radiotherapy techniques make it possible to deliver a heterogeneous dose by IMRT. FDG PET/CT can help define patients or volumes at risk of recurrence. However, Aerts et al. [9] and Calais et al. [10] showed that high FDG uptake areas on pre-therapeutic FDG PET/CT scans were highly correlated to the sites of local relapse or persistent abnormalities on post-therapeutic scans. These findings lead to consider the idea of dose-escalation on a smaller volume, allowing a better local control of the disease and minimising in parallel early and late toxicity.

The FDG PET/CT fixing per-treatment can also be a volume of interest. Per-radiotherapy FDG PET/CT can be performed without artefacts (lung inflammation) and the persistence of 42Gy fixation is very pejorative [11, 12]. As a result, we proposed a French multicenter study with dose increase on per-radiotherapy FDG volume (RTEP7, NCT02473133). Another study is also underway in the USA by the RTOG (RTOG 1106) and encouragingpreliminary results have been published by Kongs et al. in a phase 2 [13].

The definition of BTV (Biologic Target Volume) is a crucial step of treatment planning in radiation therapy. Many methods of pre-treatment segmentation have been defined but there is no segmentation method in the process of radiotherapy. For Until now, manual delineation of FDG PET positive tissues is the gold standard, despite poor reproducibility [14].

For pre-radiotherapy, many methods have been proposed in the literature. The first methods are a fixed standard uptake value (SUV), for example 2.5 [15, 16] or a threshold value around 40% of the maximum standard uptake value (SUV_max_) within the lesion [16, 17]. The last recommendations, published by the European Association of Nuclear Medicine (EANM), suggested a delineation of the MTV by applying 3D isocontours at 41% or 50% of SUV_max_ [5, 18, 19].

However, these methods are not optimal for low contrast or small volumes [20]. which can be the case on per-therapeutic PET/CT images. Thus, several complex methods have been developed [18–29]. None of them has proven its superiority yet [30]. This absence of consensus can be a problem in multicentre trials, where acquisition reproducibility is poor and devices correspond to different PET/CT models, possibly from different generation technologies. Another limitation comes from the availability of delineation softwares, especially in case of sophisticated approaches.

The aim of this article is to propose a reliable, reproducible and easy delineation method applicable in clinical routine and suitable for multicentre studies, in the specific context of per-therapeutic FDG PET/CT with potentially small volume and low contrast. This step was a prerequisite for the RTEP7 study.

## Material and methods

### Patient population and treatment

Data were extracted from 4 prospective studies corresponding to a total of 67 patients, respectively S1, S2, S3 and S4, where S1–3 correspond to monocentric clinical trials (Centre Henri Becquerel, Rouen, France) [11, 12, 31] (NCT01261598, NCT01261585) and S4 (NCT01576796) an ongoing multicentre clinical trial study [31], in which patients had given written and informed consent. All patients were treated with radiation therapy alone or concomitant chemoradiotherapy for inoperable stage II or III NSCLC. Patients were treated by conformational radiation therapy. The dose prescription corresponded to 66 Gy in 33 fractions, with 2 Gy per fraction given daily, 5 days a week. The mean age was 59 years. There were 13 women and 54 men presenting stage II (10%) or stage III (90%) NSCLC. Clinical data are summarized in Table 1.

**Table 1:** Clinical, pathological and therapeutic data

Number of patients	67
Age (years)	Mean: 59 (min 38; max 80)
Sex (number of patients)	Women: 13; Men: 54
Tumoral stage:	
- II A	2
- II B	5
- III A	25
- III B	35
Histology:	
- Adenocarcinoma	24 (5 poorly differentiated)
- Squamous cell carcinoma	37
- Undifferentiated carcinoma	6
Type of treatment:	
- Radiation therapy	21
- Concomitant radiochemotherapy	46
Dose received before per-therapeutic PET/CT	Mean: 43 Gy (min: 32 Gy; max: 52 Gy)

### PET/CT imaging

For patients treated at the Centre Henri Becquerel, the per-therapeutic PET/CT was performed on a Biograph Sensation 16 Hi-Rez device (Siemens Medical Solutions, Erlangen, Germany), without time of flight system or image reconstruction algorithm incorporating point-spread function. Forty-six patients underwent their PET/CT on this device. They were unrolled in S1–3 monocentric clinical trials (39 patients), and in S4 (7 patients). As the PET/CT device corresponded to an old generation model, these patients were grouped into a database called S_old_.

Patients who underwent their FDG PET/CT on a new generation of positron-emission tomograph came from S4 study. All the image reconstruction algorithms incorporated a point-spread function, while some of them used a time of flight system (ToF). They were grouped into a database called S_New_ (21 patients). The PET/CT models and their characteristics are listed in Appendix.

All 67 patients underwent a FDG PET/CT during the fifth week of radiation therapy. Protocols of acquisition and reconstruction followed EANM procedure guidelines [5], but they were inherent to each nuclear medicine department. On the other hand, they were the same for a given device.

### PET/CT analysis

First, per-therapeutic PET/CT scans were delineated in consensus using a Planet Onco workstation (PlanetOnco, v.2.0; DOSISoft) at the Centre Henri Becquerel (Rouen, France) by two experienced physicians of the same center: one nuclear medicine physician and one radiation oncologist with clinical practice in lung cancer. The delineation was performed using different thresholds until the volume corresponded with the one obtained by manual delineation, leading to Th_GStd_, the gold standard threshold. SUV_max_ of the lesion was also extracted leading to (Th_GStd_, SUV_max_) pairs of values.

Then, primary lung tumours (pr) were isolated from lymph nodes (no), leading to 4 classes of lesions: S_Old_(pr), S_New_(pr), S_Old_(no) and S_New_(no) lesions.

### PerPET-RT segmentation method

The graphical representation of y (Th_GStd_) as a function of x (SUVmax) showed that the shape of the curve could be approximated as the natural logarithm of the reciprocal of x.

The method proposed to easily segment the MTV on a per-therapeutic PET/CT during the fifth week of treatment of NSCLC, called perPET-RT, is based on an adaptive thresholding method according to the following expression:

*Th*_*opt*_ = *A*. ln(1/*SUV*_max_) + *B* *Eq. 1.*

where Th_opt_(%) is the optimal threshold to be applied, SUV_max_ the maximum of the SUV in the tumour (primary or node) to be segmented, and A and B, two constants depending on the kind of lesion (primary or node) leading respectively to (A_pr_, B_pr_) and (A_no_, B_no_).

One can note that Eq. 1 corresponds to a linear relationship between Th_opt_ and X = ln(1/SUV_max_), where A is the slope of the line and B the intercept, leading to the following expression:

*Th*_*opt*_ = *A*. *X* + *B* *Eq. 2.*

### Segmentation methods for performance comparison

The performance of perPET-RT was compared to several segmentation methods, based on thresholding, applied by a third experienced physician, independently from the consensual delineation used for the gold standard:

- Fixed SUV-values: 2; 2.5; 3; 3.5;
- Fixed threshold corresponding to a percentile of the maximum SUV (% of SUV_max_): 40, 50, 60, 70%;
- An adaptive thresholding method, called AOV, where the threshold to be applied corresponds to 1.5 times the mean SUV measured in an aorta volume of 1 cc [21];
- Two adaptive thresholding methods: COA and ADT [20, 22]. The two methods were calibrated according to the recommendations respectively from Schaefer et al. [20] and Vauclin et al. [23] for Biograph Sensation 16 Hi-Rez device.

### Data analysis

#### Regression function of perPET-RT

For primary tumours and nodes, the couples of values (Th_opt_, SUV_max_) were defined, as well as the associated couple of constants (i. e. (A_pr_, B_pr_) and (A_no_, B_no_)) of the linear regression (*Eq. 2*). The fits were obtained by minimizing the residuals by computing their coefficient of determination (R^2^).

The robustness of the adaptive threshold calibration procedure was evaluated by testing whether the slopes and the intercepts of the two datasets issued from the two PET models (old vs. new) were significantly different [33].

First, slopes were compared. If this first *p*-value was less than 0.05, it could be concluded that the lines were significantly different. In that case, there was no point in comparing the intercepts. Otherwise, intercepts were compared. If this second p-value was high, there was no compelling evidence that the lines were different. The software used was GraphPad Prism 5 (Version 5.0 SAS Institute Inc., CA, USA).

#### Agreement of segmented volumes

The performance of perPET-RT method was evaluated using the Dice similarity coefficient (DSC) according to the following expression:

$$ DSC=\frac{2\left(X\cap Y\right)}{\left(X\cup Y\right)} $$ *Eq. 3.*

Where X corresponds to the gold standard volume and Y the volume segmented by perPET-RT.

The agreement between the segmented volumes using other segmentation methods was also performed using DSC.

As two adaptive thresholding methods (ADT and COA) were calibrated only on the Biograph Sensation 16 Hi-Rez device, the segmentation was only done on S_old_ data for these 2 methods.

At, first a descriptive analysis of DSC was performed for each segmentation method by computing median (DSC_med_), minimum (DSC_min_) and maximum (DSC_max_) of DSC. For this analysis, first*/*third quartiles and first*/*ninth deciles of DSC-values were also extracted leading to the estimation of the inter-quartile range (difference between third and first quartiles, i.e. including 50% of the data) and the inter-decile range (difference between ninth and first deciles, i.e. including 80% of the data. Box and Whiskers plots were established. In order to compare the segmentation methods, a non-parametric analysis of DSC was performed. A *p*-value less than 0.05 was considered to be statistically significant. A Bonferonni *post-hoc* test was used.

The following criteria for the Cohen κ test were chosen to qualify the agreement of the segmentation methods: 0–0.2, poor agreement; 0.21–0.40, fair agreement; 0.41–0.60, moderate agreement; 0.61–0.80, good agreement; and 0.81–1.00, very good agreement (21).

## Results

### Per-therapeutic PET results

**Fig. 1:**
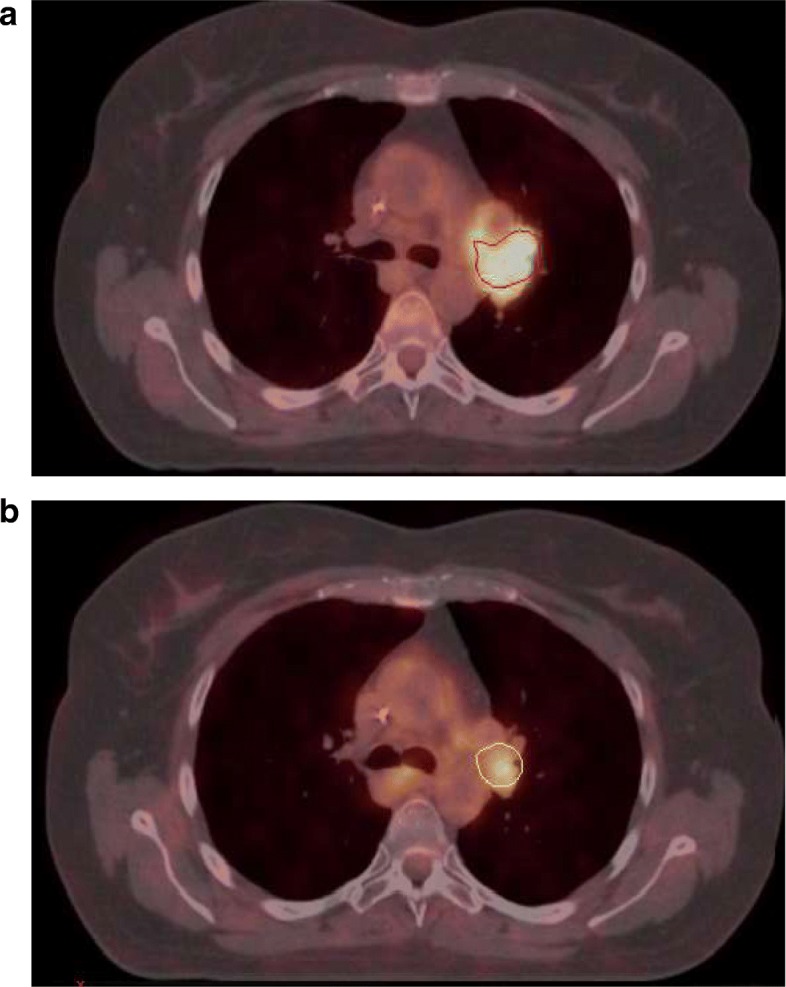
Patient with stage IIIA left lung adenocarcinoma. FDG PET/CT performed before **(a.)** and during **(b)** radiation therapy. Pre therapeutic scan **(a)** show left para-hilar hypermetabolism with SUV_max_ = 9.6 and MTV = 15.4 cc defined with a threshold value of 41% SUV_max_. Per-therapeutic data **(b)** reveals a decrease in FDG uptake (SUV_max_ = 4.2) and MTV = 4.8 cc. defined by the experts with a threshold value of 55% of SUV_max_

Patients underwent per-therapeutic PET/CT after a mean dose of 43 Gy (see Table 1). Sixty-one of the 67 patients (91%) had persistent hypermetabolic lesions on these scans, but MTV and SUV_max_ were lower on PET/CT during the treatment if compared to those of pre-therapeutic PET/CT. An example is given in Fig. 1.

A total of 111 lesions were identified: 51 lung tumours and 60 mediastinal nodes. Their main characteristics such as metabolic volume, SUV_max_ and threshold applied by the experts for delineation are reported in Table 2.

**Table 2:** Metabolic characteristics of primary tumours (pr) and involved lymph nodes (no), FDG uptake, threshold used by the experts for metabolic tumour volume delineation and corresponding volume. New and Old refer to the generation of PET device

	SUV_max_ range	SUV_max_ Mean/*Median*	Thresholds range (%SUV_max_)	Thresholds Mean/*Median* (%SUV_max_)	Volumes range (cc)	Volumes Mean/*Median* (cc)
S_Old_(pr) (*n* = 32)	2.5–14.1	6.2/*5.8*	34–66	50/*50*	0.26–65	12/*17*
S_Old_(no) (*n* = 38)	2.4–8.4	4.6/*4.6*	46–73	60/*59*	0.26–13	3/*2*
S_New_(pr) (*n* = 19)	2.5–36.5	10.5/*8.0*	16–65	43/*44*	0.47–140	20/*10*
S_New_(no) (*n* = 22)	2.6–9.3	5.4/*4.6*	44–71	57/*59*	0.57–25	4.5/*1.5*

### Regression function

**Fig. 2:**
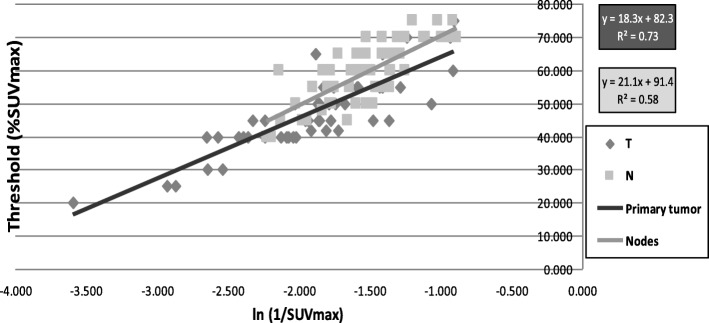
Each lesion is represented as a diamond for lung lesions and a square for involved lymph nodes. Th_opt_, the optimal threshold to be applied for delineation is expressed as a linear regression such as Th_opt_ = A.[ln (1/SUV_max_)] + B of the maximum standard uptake value (SUV_max_). The expression of Th_opt_ for lung lesions and lymph nodes are presented with their respective coefficient of determination R^2^

In Fig. 2, are given the pairs of points (Th_GStd_,Ln (1/SUV_max_)) for both primary tumours and nodes. This Fig. 2 shows also the plots corresponding to the two linear regressions (primary vs. nodes). There was no statistical difference between slopes. However, a significant difference existed between their intercepts (*p* < 0.01). On the other hand, for a given type of lesion, no significant difference was found between the lines obtained with the old and the new generation PET/CT devices.

### Agreement of segmented volumes

**Fig. 3:**
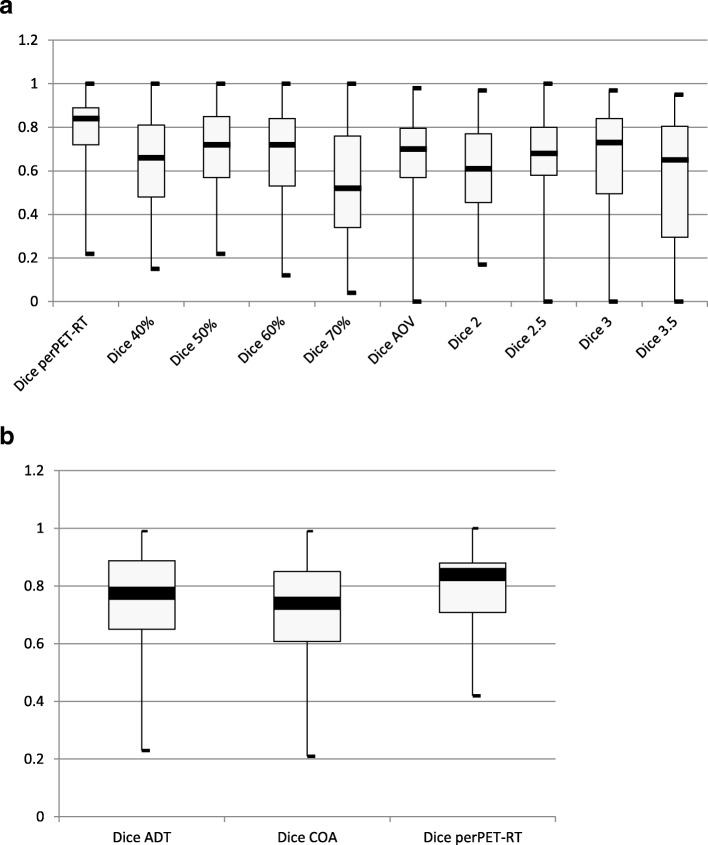
Descriptive statistics of DSC for each segmentation methods represented as Box-and-whisker plots for perPET-RT, AOV and fixed thresholding methods for the 111 lesions **(a)** and for perPET-RT, ADT and COA for the 70 lesions observed on the Biograph Hi-Rez (**b**)

In Fig. 3a are given the descriptive statistics of perPET-RT and the other segmentation methods (AOV, fixed threshold methods) using all the lesions. The segmentation with perPET-RT showed a good to a very good agreement with respect to the experts since the mean value and standard deviation of DSC were 0.78 ± 0.15 for mediastinal lymph nodes and 0.81 ± 0.13 for lung tumours. In Fig. 3b are also given the descriptive statistics of perPET-RT and ADT and COA, but only on the 70 lesions observed on the Biograph Hi-Rez device.

PerPET-RT showed a significant better agreement compared to the other segmentation methods (*p* < 0.001), except for ADT (*p* = 0.11) which showed a DSC mean value and standard deviation of 0.75 ± 0.17.

## Discussion

With this study, we propose a perPET-RT segmentation method easy to use and adapted to multicentre studies. There are no reference methods for segmentation during radiotherapy, but standard techniques may overestimate target volumes (low fixation during the treatment). The method proposed in the present study, with data extracted from 4 prospective studies, is satisfying, with good to very good agreement when compared to manual delineation during radiation therapy.

This study has several limitations. We have a limited number of patients because all patients had to have a per-radiotherapy PET/CT with persistent 42Gy fixation. If you want to increase the dose to a low volume, it is important to define it precisely and the use of 4D PET could be interesting in this context. All patients were included in studies in which 4D PET was not requested. However, the large volumes or node volumes in the case of radiochemotherapy are not very mobile.

With this method, only one dose level is possible but dose painting techniques could be interesting with heterogeneous doses depending on the FDG fixation. The dose painting could take into account the dose in relation to the FDG fixation but also the dose to be delivered to organs at risk, particularly for lymph node fixation.

Despite combination of chemotherapy and radiation therapy, survival rates remain poor for stage III NSCLC [34, 35]. Patients with locally advanced NSCLC have a very high risk of relapse and/or progression leading to death within the year if they express high metabolic profiles on a per-therapeutic PET/CT scans performed during the fifth week of radiation therapy [12]. The dose-escalation on a smaller volume delineated on the per-therapeutic PET/CT is aiming for a better local control of the disease and to avoid exacerbated early and late toxicity. Nevertheless, this concept is altered the lack of available FDG PET segmentation methods in clinical routine adapted to per-therapeutic FDG PET/CT (around 43 Gy). A randomized phase II dose-escalation trial demonstrated the feasibility of significant dose-escalation on the primary tumour or the high FDG uptake subvolume of the primary tumour without violating the dose constraints for the organs at risk [36]. Dose-escalation planning based on interim FDG PET/CT scans (around 50 Gy of radiation therapy) is feasible, but none of the semi-automatic segmenting tools (including threshold of 2.5, 40% of SUV_max_ or AOV method) seemed reliable to define volumes correctly [37]. All the methods developed have essentially been developed for tumors more than for lymph node fixation and for tumors before treatment. Radiation therapy modifies the tumor to background ratio. In our study, there was a good agreement between the different methods, but the perPET-RT method had the best agreement with the experts. The ADT method was not significantly different from our method, this is probably due to the fact that it was developed specifically for lung cancers as in our case.

In addition, ongoing clinical trials are evaluating the impact of dose escalation on progression-free survival and overall survival. One of them [32] proposes to increase the dose to hypoxic tumoral areas. Another current clinical trial, lead by the RTOG group, seeks to determine if the dose to the tumour can be increased when a personalized radiation treatment is planned with a PET/CT scan acquired at 40–46 Gy of radiotherapy in patients with inoperable or unresectable stage III NSCLC. The method used for tumoral volume delineation corresponds to the AOV method [38].

PerPET-RT is one of the thresholding-based approaches which are the most widely available techniques in clinical routine. It requires knowing the type of lesion (primary or node) and the measurement of SUV_max_, which is, in practice, easier and more reproducible than thresholding methods based on contrast (COA and ADT) or a mean SUV measurement (AOV). Another advantage of perPET-RT is that there is no need to calibrate the method on PET/CT models, unlike many adaptive thresholding methods. In our database, the method was not sensitive to the generation of PET models. This result has to be confirmed on other databases. Nevertheless, the concept of using such an approach in clinical routine or in mono or multicentre clinical trials is possible and easy to implement. As gold standard, a consensus threshold value was used. Palie et al. showed that there was an excellent reproducibility in delineation of MTVs by the physicians [39]**.** In addition, a recently published study demonstrated the added value of consensus methods in delineation [23]. PerPET-RT was compared with other thresholding methods (fixed or adaptive) due to the ease of use of these techniques. On the other hand, more sophisticated algorithms were not used due to the lack of availability in the context of multicenter clinical trials.

The clinical impact of dose-escalation on the volumes defined by this method is yet to be evaluated by a recently started multicentre clinical trial [40].

## Conclusion

PerPET-RT, a thresholding-based approach, was proposed and validated on 4 prospective studies. We have showed that this method is reliable, easy to use and accurate for tumoral delineation on per-therapeutic FDG PET/CT. This method may be used to delineate tumoral volumes for dose-escalation planning. A clinical trial evaluating the impact of dose-escalation radiation therapy in NSCLC has already started in France (PHRC 2014, IFCT 1402-RTEP7).

## Appendix

**Table 3:** New generation PET/CT devices with their respective system characteristics and the number of patients per device

Type of PET/CT device	Constructor	Number of patients and lesions (tumor, node)	Reconstruction algorithm with point-spread function	ToF system
Gemini TF	Philips	3 (2, 4)	Yes	Yes
GE 690	General Electrics	8 (8, 12)	Yes	Yes
Biograph mCT 40	Siemens	4 (3, 2)	Yes	Yes
Biograph 6	Siemens	4 (4, 1)	Yes	No
Biograph mCT	Siemens	2 (2, 3)	Yes	Yes

## Abbreviations

FDG: fluorine 18 fluorodeoxyglucose
MTV: metabolic tumour volume
NSCLC: non-small cell lung cancer
PET/CT: positron emission tomography/computed tomography
SUV_max_: the maximum standard uptake value

## References

[1] PDQ Adult Treatment Editorial Board (2002). Non-small cell lung Cancer treatment (PDQ®): health professional version. PDQ Cancer information summaries.

[2] Albain KS, Swann RS, Rusch VW, Turrisi AT, Shepherd FA, Smith C (2009). Radiotherapy plus chemotherapy with or without surgical resection for stage III non-small-cell lung cancer: a phase III randomised controlled trial. Lancet Lond Engl.

[3] Goldstraw P, Ball D, Jett JR, Le Chevalier T, Lim E, Nicholson AG (2011). Non-small-cell lung cancer. Lancet Lond Engl.

[4] Jarritt P H, Carson K J, Hounsell A R, Visvikis D (2006). The role of PET/CT scanning in radiotherapy planning. The British Journal of Radiology.

[5] Boellaard R, Delgado-Bolton R, Oyen WJG, Giammarile F, Tatsch K, Eschner W (2015). FDG PET/CT: EANM procedure guidelines for tumour imaging: version 2.0. Eur J Nucl Med Mol Imaging.

[6] van Baardwijk A, Bosmans G, Boersma L, Buijsen J, Wanders S, Hochstenbag M (2007). PET-CT-based auto-contouring in non-small-cell lung cancer correlates with pathology and reduces interobserver variability in the delineation of the primary tumor and involved nodal volumes. Int J Radiat Oncol Biol Phys.

[7] Schreurs LMA, Busz DM, Paardekooper GMRM, Beukema JC, Jager PL, Van der Jagt EJ (2010). Impact of 18-fluorodeoxyglucose positron emission tomography on computed tomography defined target volumes in radiation treatment planning of esophageal cancer: reduction in geographic misses with equal inter-observer variability: PET/CT improves esophageal target definition. Dis Esophagus Off J Int Soc Dis Esophagus ISDE.

[8] Bradley JD, Paulus R, Komaki R, Masters G, Blumenschein G, Schild S (2015). Standard-dose versus high-dose conformal radiotherapy with concurrent and consolidation carboplatin plus paclitaxel with or without cetuximab for patients with stage IIIA or IIIB non-small-cell lung cancer (RTOG 0617): a randomised, two-by-two factorial phase 3 study. Lancet Oncol.

[9] Aerts HJWL, van Baardwijk AAW, Petit SF, Offermann C, van LJ, Houben R (2009). Identification of residual metabolic-active areas within individual NSCLC tumours using a pre-radiotherapy (18) Fluorodeoxyglucose-PET-CT scan. Radiother Oncol J Eur Soc Ther Radiol Oncol.

[10] Calais J, Thureau S, Dubray B, Modzelewski R, Thiberville L, Gardin I (2015). Areas of high 18F-FDG uptake on preradiotherapy PET/CT identify preferential sites of local relapse after chemoradiotherapy for non-small cell lung cancer. J Nucl Med Off Publ Soc Nucl Med.

[11] Edet-Sanson A, Dubray B, Doyeux K, Back A, Hapdey S, Modzelewski R (2012). Serial assessment of FDG-PET FDG uptake and functional volume during radiotherapy (RT) in patients with non-small cell lung cancer (NSCLC). Radiother Oncol J Eur Soc Ther Radiol Oncol.

[12] Vera P, Mezzani-Saillard S, Edet-Sanson A, Ménard J-F, Modzelewski R, Thureau S (2014). FDG PET during radiochemotherapy is predictive of outcome at 1 year in non-small-cell lung cancer patients: a prospective multicentre study (RTEP2). Eur J Nucl Med Mol Imaging.

[13] Kong FM, Ten Haken RK, Schipper M, Frey KA, Hayman J, Gross M (2017). Effect of Midtreatment PET/CT-adapted radiation therapy with concurrent chemotherapy in patients with locally advanced non-small-cell lung Cancer: a phase 2 clinical trial. JAMA Oncol.

[14] Hatt M, Visvikis D. Defining radiotherapy target volumes using 18**F**-fluoro-deoxy-glucose positron emission tomography/computed tomography: still a Pandora’s box?: in regard to Devic et al. (Int J Radiat Oncol Biol Phys 2010). Int J Radiat Oncol Biol Phys. 2010;78(5):1605. 10.1016/j.ijrobp.2010.08.002 21092836

[15] Paulino AC, Johnstone PAS (2004). FDG-PET in radiotherapy treatment planning: Pandora’s box?. Int J Radiat Oncol Biol Phys.

[16] Nestle U, Schaefer-Schuler A, Kremp S, Groeschel A, Hellwig D, Rübe C (2007). Target volume definition for 18F-FDG PET-positive lymph nodes in radiotherapy of patients with non-small cell lung cancer. Eur J Nucl Med Mol Imaging.

[17] Erdi YE, Mawlawi O, Larson SM, Imbriaco M, Yeung H, Finn R (1997). Segmentation of lung lesion volume by adaptive positron emission tomography image thresholding. Cancer.

[18] Boellaard R, Krak NC, Hoekstra OS, Lammertsma AA (2004). Effects of noise, image resolution, and ROI definition on the accuracy of standard uptake values: a simulation study. J Nucl med off Publ Soc. Nucl Med.

[19] Krak NC, Boellaard R, Hoekstra OS, Twisk JWR, Hoekstra CJ, Lammertsma AA (2005). Effects of ROI definition and reconstruction method on quantitative outcome and applicability in a response monitoring trial. Eur J Nucl Med Mol Imaging.

[20] Doyeux K, Vauclin S, Hapdey S, Daouk J, Edet-Sanson A, Vera P (2013). Reproducibility of the adaptive thresholding calibration procedure for the delineation of 18F-FDG-PET-positive lesions. Nucl Med Commun may.

[21] Schaefer A, Kremp S, Hellwig D, Rübe C, Kirsch C-M, Nestle U (2008). A contrast-oriented algorithm for FDG-PET-based delineation of tumour volumes for the radiotherapy of lung cancer: derivation from phantom measurements and validation in patient data. Eur J Nucl Med Mol Imaging.

[22] Thureau S, Chaumet-Riffaud P, Modzelewski R, Fernandez P, Tessonnier L, Vervueren L (2013). Interobserver agreement of qualitative analysis and tumor delineation of 18F-fluoromisonidazole and 3′-deoxy-3’-18F-fluorothymidine PET images in lung cancer. J Nucl Med Off Publ Soc Nucl Med.

[23] Schaefer A, Vermandel M, Baillet C, Dewalle-Vignion AS, Modzelewski R, Vera P (2016). Impact of consensus contours from multiple PET segmentation methods on the accuracy of functional volume delineation. Eur J Nucl Med Mol Imaging.

[24] Vauclin S, Doyeux K, Hapdey S, Edet-Sanson A, Vera P, Gardin I (2009). Development of a generic thresholding algorithm for the delineation of 18FDG-PET-positive tissue: application to the comparison of three thresholding models. Phys med biol.

[25] Geets X, Lee JA, Bol A, Lonneux M, Grégoire V (2007). A gradient-based method for segmenting FDG-PET images: methodology and validation. Eur J Nucl Med Mol Imaging.

[26] Onoma DP, Ruan S, Thureau S, Nkhali L, Modzelewski R, Monnehan GA (2014). Segmentation of heterogeneous or small FDG PET positive tissue based on a 3D-locally adaptive random walk algorithm. Comput Med Imaging Graph Off J Comput Med Imaging Soc dec.

[27] Belhassen S, Zaidi H (2010). A novel fuzzy C-means algorithm for unsupervised heterogeneous tumor quantification in PET. Med Phys mar.

[28] Dewalle-Vignion A-S, Betrouni N, Lopes R, Huglo D, Stute S, Vermandel M (2011). A new method for volume segmentation of PET images, based on possibility theory. IEEE Trans Med Imaging.

[29] Hatt M., Cheze le Rest C., Turzo A., Roux C., Visvikis D. (2009). A Fuzzy Locally Adaptive Bayesian Segmentation Approach for Volume Determination in PET. IEEE Transactions on Medical Imaging.

[30] Zaidi H, El Naqa I (2010). PET-guided delineation of radiation therapy treatment volumes: a survey of image segmentation techniques. Eur J Nucl Med Mol Imaging.

[31] Vera P, Bohn P, Edet-Sanson A, Salles A, Hapdey S, Gardin I (2011). Simultaneous positron emission tomography (PET) assessment of metabolism with 18F-fluoro-2-deoxy-d-glucose (FDG), proliferation with 18F-fluoro-thymidine (FLT), and hypoxia with 18fluoro-misonidazole (F-miso) before and during radiotherapy in patients with non-small-cell lung cancer (NSCLC): a pilot study. Radiother Oncol jan.

[32] Radiotherapy Dose Complement in the Treatment of Hypoxic Lesions Patients With Stage III Non-small-cell Lung Cancer - ClinicalTrials.gov. https://clinicaltrials.gov/ct2/show/NCT01576796?term=rtep&rank=6

[33] Zar J (1984). Biostatiscal analysis.

[34] Aupérin A, Le Péchoux C, Rolland E, Curran WJ, Furuse K, Fournel P (2010). Meta-analysis of concomitant versus sequential radiochemotherapy in locally advanced non-small-cell lung cancer. J Clin Oncol Off J Am Soc Clin Oncol.

[35] Curran WJ, Paulus R, Langer CJ, Komaki R, Lee JS, Hauser S (2011). Sequential vs. concurrent chemoradiation for stage III non-small cell lung cancer: randomized phase III trial RTOG 9410. J Natl Cancer Inst.

[36] van Elmpt W, De Ruysscher D, van der Salm A, Lakeman A, van der Stoep J, Emans D (2012). The PET-boost randomised phase II dose-escalation trial in non-small cell lung cancer. Radiother Oncol J Eur Soc Ther Radiol Oncol.

[37] Kelsey CR, Christensen JD, Chino JP, Adamson J, Ready NE, Perez BA (2016). Adaptive planning using positron emission tomography for locally advanced lung cancer: a feasibility study. Pract Radiat Oncol.

[38] Study of Positron Emission Tomography and Computed Tomography in Guiding Radiation Therapy in Patients With Stage III Non-small Cell Lung Cancer - ClinicalTrials.govhttps://clinicaltrials.gov/ct2/show/NCT01507428?term=rtog+1106&rank=1.

[39] Palie O, Michel P, Ménard J-F, Rousseau C, Rio E, Bridji B (2013). The predictive value of treatment response using FDG PET performed on day 21 of chemoradiotherapy in patients with oesophageal squamous cell carcinoma. A prospective, multicentre study (RTEP3). Eur J Nucl Med Mol Imaging.

[40] Study of Interest of Personalized Radiotherapy Dose Redistribution in Patients With Stage III NSCLC - ClinicalTrials.govhttps://clinicaltrials.gov/ct2/show/NCT02473133?term=lung+rouen+pet&rank=6

